# An evaluation of the quality of ear health services for Aboriginal children living in remote Australia: a cascade of care analysis

**DOI:** 10.1186/s12913-023-10152-z

**Published:** 2023-10-31

**Authors:** Jiunn-Yih Su, Amanda Jane Leach, Alan Cass, Peter Stanley Morris, Kelvin Kong

**Affiliations:** 1 Menzies School of Health Research, Charles Darwin University, Darwin, NT Australia; 2 Royal Darwin Hospital, Darwin, NT Australia; 3 John Hunter Children’s Hospital, Newcastle, NSW Australia

**Keywords:** Otitis media, Ear health services, Cascade of care analysis, Aboriginal and Torres Strait Islander children

## Abstract

**Background:**

In the Northern Territory (NT) the prevalence of otitis media (OM) in young Aboriginal children living in remote communities has persisted at around 90% over the last few decades. OM-associated hearing loss can cause developmental delay and adversely impact life course trajectories. This study examined the 5-year trends in OM prevalence and quality of ear health services in remote NT communities.

**Methods:**

A retrospective analysis was performed on de-identified clinical data for 50 remote clinics managed by the NT Government. We report a 6-monthly cascade analysis of the proportions of children 0–16 years of age receiving local guideline recommendations for surveillance, OM treatment and follow-up at selected milestones between 2014 and 2018.

**Results:**

Between 6,326 and 6,557 individual children were included in the 6-monthly analyses. On average, 57% (95%CI: 56–59%) of eligible children had received one or more ear examination in each 6-monthly period. Of those examined, 36% (95%CI: 33–40%) were diagnosed with some type of OM, of whom 90% had OM requiring either immediate treatment or scheduled follow-up according to local guidelines. Outcomes of treatment and follow-up were recorded in 24% and 23% of cases, respectively. Significant decreasing temporal trends were found in the proportion diagnosed with any OM across each age group. Overall, this proportion decreased by 40% over the five years (from 43 to 26%).

**Conclusions:**

This cascade of care analysis found that ear health surveillance and compliance with otitis media guidelines for treatment and follow-up were both low. Further research is required to identify effective strategies that improve ear health services in remote settings.

## Introduction

Otitis media (OM) is a serious public health problem in Australian Aboriginal and Torres Strait Islander (hereafter respectfully referred to as Aboriginal) children [1, 2]. Compared with their non-Aboriginal counterparts, Aboriginal children are affected by OM earlier in life, more frequently, persistently and severely with more serious complications [3–6]. The commonest complication of OM, conductive hearing loss, has been shown in longitudinal cohort studies to affect Aboriginal children throughout the first three years of life [7, 8]. As this early childhood period is a critical period for cognitive and language development, hearing loss suffered during this period can affect this development and in turn adversely affect children’s later life course trajectories [5]. Recent studies conducted in remote Australian communities using linked administrative data have shown that OM-associated hearing impairment in Aboriginal children negatively impacts on early childhood development, school attendance and academic performance, and increases the risk of child maltreatment and involvement with the youth justice system [9–14].

Located in the northern and central parts of Australia, the Northern Territory (NT) has the smallest population (about 246,500 in 2020) but the highest proportion of Aboriginal people (~ 30%) of all Australian states and territories. Past cross-sectional surveys conducted in NT remote communities had reported prevalence of OM among Aboriginal children between 68 and 91% [2, 15–17]. Although a number of public health and clinical interventions had been implemented in the last few decades, including universal childhood pneumococcal vaccination programs and evidence-based clinical guidelines, a recent local study still found OM prevalence persisting at around 90% in children 6–36 months of age [2]. Recent local studies also found a decline in the most concerning type of OM, chronic suppurative otitis media (CSOM), counteracted by a higher prevalence of other forms of otitis media (OM with effusion, OME, and acute OM, AOM), and no increase in prevalence of children without OM [2, 15]. The consecutive introduction of the 7-valent pneumococcal conjugate vaccine (PCV7, in 2001) and the 10-valent pneumococcal *Haemophilus influenzae* protein D-conjugate vaccine (PHiD-CV10, replacing PCV7 in 2009) was associated with a decline in the prevalence of tympanic membrane perforation (TMP) from 24% (in 2001, prior to vaccine introduction) to 17% (in 2008, PCV7 era), and then 14% (in 2010, PHiD-CV10 era) [15, 18]. The subsequent switch to 13-valent pneumococcal conjugate vaccine (PCV13) in 2011 did not further reduce the prevalence of TMP (12% in 2013) [2].

Considering the positive but limited response of OM prevalence to the interventions implemented, namely otitis media guidelines and PCVs, the attention in recent years has turned to improving the quality and consistency of ear health services at the primary care settings. Well-coordinated active ear programs within Primary Health Care such as scheduled ear checks for early detection of OM (which is predominantly asymptomatic in this population), and guideline-compliant management have potential to prevent chronicity, progression, and severity of disease, and therefore hearing loss and reduce risk of social disadvantage [7, 19, 20].

The Hearing for Learning Initiative (HfLI) has been designed and conducted as a stepped-wedge cluster randomised trial to test if specifically trained, community-based ear health facilitators (EHFs) can improve the delivery of ear and hearing health services in remote settings in the NT [21]. The current study was conducted as part of the HfLI to document pre-intervention assessment of the NT Government-run primary care services for ear health with regards to the key primary and secondary outcomes of program. The results will be used as the baseline statistics in the evaluation of the effectiveness of the interventions proposed by the HfLI.

## Materials and methods

### Study design and setting

This was a retrospective analysis of de-identified unit-record medical record data for ear health from 50 remote NT Aboriginal communities. The dataset accounts for approximately half of all remote communities in the NT. In each of these communities, health care was provided by one single community clinic managed by the NT Health. The period of the study covered calendar years 2014–2018.

### Participants and data sources

Research data were retrieved from the centralised electronic clinical information system (Primary Care Information System, PCIS) used across all NT Government-managed clinics. The children were Aboriginal children aged 0–16 years (at the time of clinic consultation) who were recorded in PCIS as usual residents of the included communities. Records for the same individual were linked with a research identification number uniquely and randomly generated by the data custodian based on the unique client identifier used in PCIS (the hospital record number, HRN). Children aged 0–16 years who were recorded as usual residents of a community in a calendar year were the study population for the same year (denominators) for calculation of proportions.

### The cascade of care analysis

We applied the ‘cascade of care’ framework to assess the quality of primary care services for ear health. This framework was originally used in evaluating the quality of HIV care delivery along its whole spectrum from testing, through diagnosis, access, retention in care and treatment, and finally to viral suppression, for the purpose of achieving the ultimate goal of improving survival and reducing transmission [22]. The ‘cascade of care’ framework is useful in identifying gaps in existing services and assisting service providers in tailoring interventions to address the identified gaps. It has since been used in a number of infectious diseases, such as latent tuberculosis [23], hepatitis B [24], hepatitis C [25], bacterial sexually transmitted infections (e.g. chlamydia [26] and syphilis [27]). In this study, we applied this analysis in order to examine a wider spectrum of ear health services, from screening ear examination, diagnosis, treatment to follow-up.

**Fig. 1:**
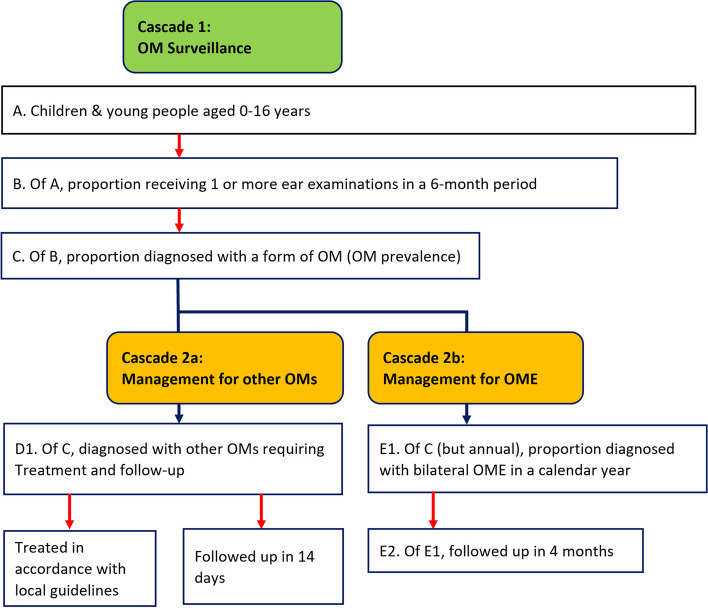
A cascade analysis of the ear health services provided at the remote clinics managed by the Northern Territory Government, 2014–2018

The ‘cascade of care’ framework used in this study consisted of two cascades (Fig. 1). Cascade 1 focused on *OM surveillance* and assessed the frequencies and results of ear examinations. Given the early onset and high prevalence of asymptomatic OM, in the study cohort as described above, regular ear examinations are crucial to early detection of OM for individuals and accurate and close monitoring of prevalence and incidence for the population. The denominator for each calendar year was the number of children up to 17 years of age recorded as residents of the community in PCIS. We then calculated the proportions of these children who had received one or more ear examinations during each 6-month period (Jan-Jun or Jul-Dec), then the proportion of children examined who had a diagnosis of OM. This proportion represented the period prevalence of OM for the study cohort.

Cascade 2 focused on the *OM management* of individuals in Cascade 1 who were diagnosed with any form of OM and required treatment or follow-up. According to local treatment guidelines, children diagnosed with otitis media with effusion (OME) need to be followed up in 3 months while those with acute OM with or without perforation or chronic suppurative OM require more immediate follow-up (generally in 7 days or shorter) [28]. Therefore, we divided Cascade 2 into two arms with Cascades 2a and 2b assigned to those diagnosed with other types of OM than OME (either AOM with or without perforation, or CSOM) and those with OME, respectively. For Cascade 2a, we calculated, within each 6-month period, the proportion of children diagnosed with other types of OM than OME who were treated in accordance with local guidelines, although we extended the proportion followed up to within 14 days, to allow for pragmatic approach to follow-up. For Cascade 2b, we calculated the proportion of children diagnosed with OM in a calendar year who were diagnosed with bilateral OME and then the proportion of those who were followed up within 4 months, which allows an extra month to the recommended 3 months.

The search for records of ear examinations was conducted using the service items in PCIS entitled "Ear Examination" and " Ear Examination—HSAK Check" (HSAK is the abbreviation for the Healthy School Age Kids program [29]). We conducted the search for records of ear examination, diagnosis and follow-up in the Results module of PCIS. The search for treatment records was done in the Medchart module using the antibiotic names (but not dose, frequency or duration) specified in the local standard treatment manual [28]. In remote NT clinics, key clinicians who made the diagnosis for middle ear conditions investigated in this study included doctors, nurses and Aboriginal Health Practitioners.

### Statistical analysis

Descriptive statistical analysis was conducted on demographic features and selected primary and secondary outcome measures of the HfLI. The details of calculation of outcome measures are listed in Table 1. We calculated all proportions per each six-month period together with their means and 95% confidence intervals (CI), except the proportions in Cascade 2b, which were calculated per calendar year. Analysis was also performed on variables such as NT geographic region (i.e. Central Australia vs. Top End). The tropical ‘Top End’ region is located in the northern third of the NT and comprises the Darwin, East Arnhem and Katherine districts. The arid ‘Central Australia’ region is located in the southern two-thirds and consists of the Barkly and Alice Springs districts. The Joinpoint Regression Program (version 4.9.0.0) was used to examine trends in all outcome measures overall and by age groups over the ten 6-month periods [30]. This program divides the time period under examination into a number of continuous linear time periods, and identifies the best fitting piecewise continuous log-linear model. The program then reports the average annual percentage changes (AAPC) for the entire period. Where relevant, we report AAPC with 95% confidence intervals (CIs) and the associated *p* value. All other analyses were conducted using Stata for Windows, Version 14 (StataCorp 2014). Statistical significance was identified by a 2-sided *p* value < 0.05 or when the 95% CI did not include the null value.

**Table 1:** Denominators and numerators used in calculating the selected outcome measures

Cascade	Step	Outcome measure	Denominator	Numerator
1	B	Proportion of those aged < 17 who received 1 or more ear examinations in a six-month period (the primary outcome of HfLI)^a^	The number of participants who were usual residents of the included communities aged < 17 at the start of the calendar year	The number of participants in the denominator who had received 1 or more ear examinations during the 6-month period under examination
	C	Proportion of those who received 1 or more ear examinations and were diagnosed with a form of otitis media	Numerator for B	The number participants who were diagnosed with a form of otitis media during the 6-month period under examination
2a	D1	Proportion of participants in C who required more immediate treatment and follow-up	Numerator for C	The number of participants who were diagnosed with a form of otitis media requiring more immediate treatment and follow-up, in the same 6-month period
	D2	Proportion of participants in D1 who were treated according to the Guidelines^b^	Numerator for D1	The number of participants in E1, who were treated according to the Guidelines^b^
	D3	Proportion of participants in E1 who were followed up in < 14 days	Numerator for D1	The number of participants in D1 who were followed up in < 14 days
2b	E1	Proportion of participants diagnosed with a form of OM, which was bilateral OME, in a calendar year	The number of participants who were usual residents of the included communities aged < 17 at the start of the calendar year, and were diagnosed with a form of OM in a calendar year	The number of participants who were diagnosed with bilateral OME in the same calendar year
	E2	Proportion of participants in 2b-E1 who were followed up in < 4 month	Numerator for E1	The number of participants who were diagnosed with bilateral OME and were followed up in < 4 month in the same calendar year

The measures in shaded cells (Cascade 2b) were calculated by calendar years. *OM *Otitis media, *OME *Otitis media with effusion, *HfLI *Hearing for Learning Initiative

^a^Two service items in the Results module of the PCIS dataset were used to identify ear examinations performed, including’EAR EXAMINATION’ and ‘EAR EXAMINATION—HSAK CHECK’ (HSAK stands for Healthy school-age kids program)

^b^Recommendations for Clinical Care Guidelines on the Management of Otitis Media in Aboriginal & Torres Strait Islander Populations (April 2010), Commonwealth of Australia

### Ethics consideration

Ethics approval to conduct this study was obtained from Human Research Ethics Committee of the NT Department of Health and the Menzies School of Health Research (#2018–3264) and Central Australian Human Research Ethics Committee (#CA-19–3308).

## Results

### Demographic features

The study population was estimated to be 6,440 children and young people aged 0–16 years (95% CI: 6331–6549) during the study period. There were significantly more males (*n* = 3280, 95% CI: 3208–3351) than females (*n* = 3160, 95% CI: 3123–3198, *p* = 0.004). The age distribution also differed significantly between male and female (Chi-squared test, *p* < 0.0005) with females having a higher proportion in the 10–16 years age group but lower in the under 2-year-old age group. No significant difference was found in the sex distribution across the five years (*p* = 0.979) or between regions (*p* = 0.287, Table 2).

**Table 2:** Demographic features of the participants, stratified by sex (CI: confidence interval)

Variable	All	Females	Males	*p* value
Number of participants				
Mean	6439.8	3160.4	3279.4	0.004
95% CI	6330.5–6549.1	3122.5–3198.3	3207.6–3351.2	
Number of participants by year				0.979
2014	6326	3122	3204	
2015	6385	3138	3247	
2016	6465	3174	3291	
2017	6466	3169	3297	
2018	6557	3199	3358	
Age group				< 0.0005
< 2	15.8%	15.1%	16.6%	
2–4	18.3%	17.8%	18.7%	
5–9	29.7%	29.7%	29.7%	
10–16	36.2%	37.4%	35.0%	
Region				
Central Australia	30.2%	30.5%	29.9%	0.287
Top End	69.8%	69.5%	70.1%	

### Ear health outcomes

#### The ‘cascade of care’ analysis

**Fig. 2:**
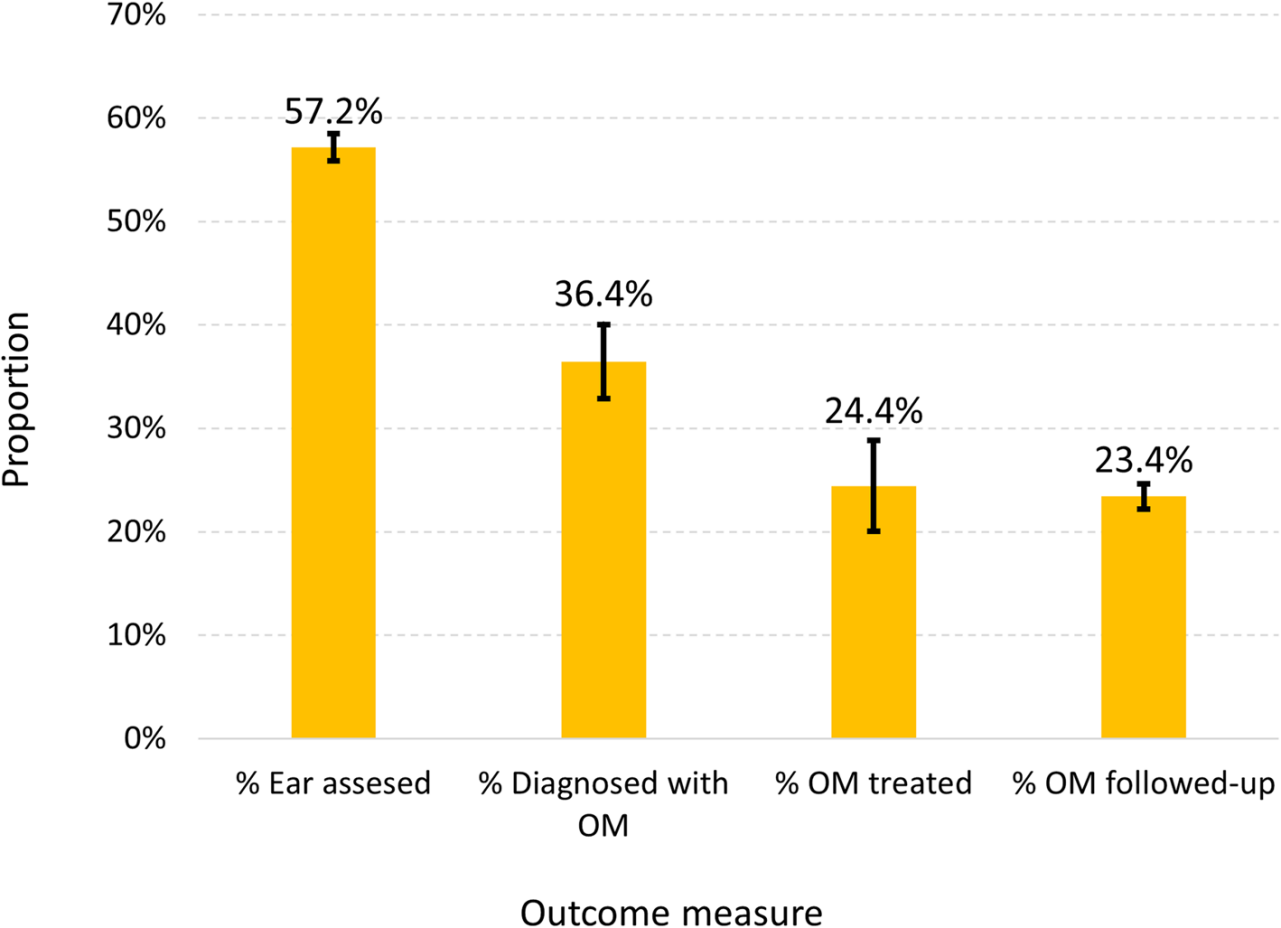
Summary results of the cascade analysis of the outcome measures (error bars indicate 95% confidence intervals, OM: otitis media)

In Cascade 1, on average, the proportion of children who had received one or more ear examinations in a 6-month period (OM surveillance) was 57.2% (95% CI: 55.9–58.5%, Fig. 2). Of those examined, 36.4% (95% CI: 32.8–40.0%) were diagnosed with some form of OM (OM prevalence). In Cascade 2a, almost 90% (89.9%, 95% CI: 88.2–91.5%) of children had OM requiring more immediate treatment and follow-up. Of those requiring immediate treatment and follow-up, fewer than 1 in 4 were treated (24.4%, 95%CI: 20.0–28.8%) or followed up (23.4%, 95%CI: 22.2–24.7%) within 14 days. Statistics for Cascade 2b were calculated by calendar year. Of children diagnosed with some form of OM, 12.1% (95% CI 8.7–15.6%) were diagnosed with bilateral OME. Of those with bilateral OME, 52.2% (95%CI: 47.2–57.2%) were followed up within 4 months.

#### Age-specific temporal trends in surveillance, prevalence, treatment, and follow-up

**Fig. 3:**
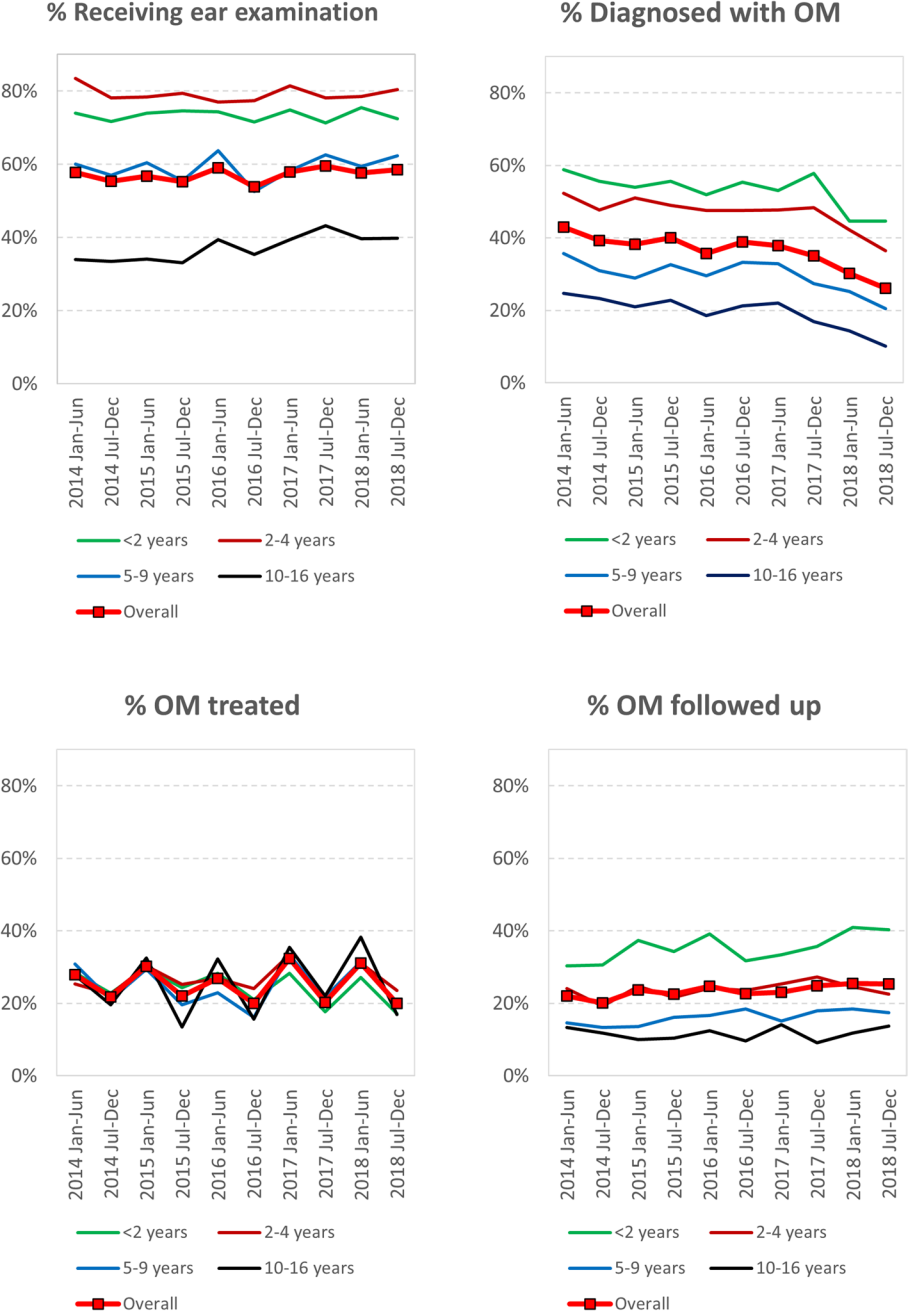
Age-specific results of outcome measures per 6-month period

The proportion of children who had received ear examinations in a 6-month period (OM surveillance) was consistently highest in those under 5 years of age (< 2 and 2–4 year old age groups) at 70% to 80%, falling to ~ 60% in the 5–9 year old age group, then 33% to 43% in the 10–16 year old age group. The overall proportion of children receiving a 6-monthly ear examination stayed just under 60% across the study period (Fig. 3).

The proportion of examined children who were diagnosed with a form of OM (OM prevalence) showed a significant decreasing trend during the study period across all age groups. Overall, prevalence decreased from 43.0% in Jan-Jun 2014 to 26.0% in Jul-Dec 2018, a substantial 40% decrease during the study period. OM prevalence was consistently highest in the under 2 (~ 55%) and 2 to 5 (~ 50%) age groups and lowest (~ 20%) in the 10–16 years old age group (Fig. 3).

The proportion of examined children who were diagnosed with OM and treated in accordance with the local guidelines (OM treatment) did not differ between age groups or change significantly over time, fluctuating between 20 to 30% across the whole study period (Fig. 3). Overall, the follow-up of children with OM within 14 days was between 22.0% in Jan-Jun 2014 and 25.4% in Jul-Dec 2018, and was consistently highest (30% to 40%) in the under 2 year old age group and lowest in the 10–16 year old age group (fluctuating between 10 and 13%) (Fig. 3).

The overall and age-specific annual percentage changes (AAPC) in OM surveillance, OM prevalence, OM treatment, and OM follow-up are detailed in Table 3. There was no evident overall temporal trend in OM surveillance (AAPC = 0.42%, 95% CI: -0.37% ~ 1.22%, *p* = 0.253). However there was an approximate overall 5% decrease per year in OM prevalence (AAPC = -5.03%, 95% CI: -8.05% ~ -1.92%, *p* = 0.002) and a small but significant increasing trend in OM follow-up within 14 days over the study period (AAPC = 1.95%, 95% CI, 0.68% ~ 3.23%, *p* = 0.007), despite no substantial increase in OM treatment according to guidelines (AAPC = -1.01%, 95%CI, -6.04 ~ 4.28%, *p* = 0.664) (Table 3).

**Table 3:** Age specific and overall results of outcome measures and trend analysis

Age group	% Received ear examination in a 6-month period			% Diagnosed with OM			% OM treated according to Guidelines			% OM followed up in < = 14 days		
	**AAPC**	**95% CI**	***P* value**	**AAPC**	**95% CI**	***P* value**	**AAPC**	**95% CI**	***P* value**	**AAPC**	**95% CI**	***P* value**
Overall	0.42	(-0.37 ~ 1.22)	0.253	-5.03	(-8.05 ~ -1.92)	0.002	-1.01	(-6.04 ~ 4.28)	0.664	1.95	(0.68 ~ 3.23)	0.007
< 2 years	-0.03	(-0.59 ~ 0.53)	0.896	-2.26	(-4.04 ~ -0.45)	0.021	-3.45	(-7.83 ~ 1.13)	0.119	2.52	(0.27 ~ 4.82)	0.032
2–4 years	-0.14	(-0.80 ~ 0.53)	0.639	-3.46	(-5.45 ~ -1.43)	0.001	0.29	(-3.73 ~ 4.48)	0.875	1.28	(-1.10 ~ 3.72)	0.252
5–9 years	0.45	(-1.10 ~ 2.03)	0.523	-4.62	(-8.75 ~ -0.30)	0.036	-0.83	(-7.17 ~ 5.95)	0.779	3.16	(1.03 ~ 5.34)	0.009
10–16 years	2.62	(1.12 ~ 4.15)	0.004	-8.35	(-12.19 ~ -4.34)	< 0.001	0.26	(-9.36 ~ 10.91)	0.953	0.27	(-3.82 ~ 4.53)	0.886

*AAPC* Average annual percentage change, *OM* Otitis media, *CI* Confidence interval. Data for AAPC and the 95%CI are changes in percentage

There were some age-specific trends, such as OM surveillance, which increased over time only in the 10–16-year-old age group (AAPC = 2.6%, 95%CI, 1.12% ~ 4.15, *p* = 0.004). Significant annual reductions in OM prevalence were seen in all age groups, predominantly in the 10–16-year-old group (-8.35%, 95%CI, -12.19 to -4.34. *p* < 0.001). Annual average percentage changes in OM treatment declined by 3.45% annually in the < 2-year-old group (95%CI -7.83 to 1.13, *p* = 0.119). OM follow-up within 14 days increased significantly in two age groups; the under 2 year olds (AAPC = 2.52%, 95%CI 0.27 to 4.82, *p* = 0.032) and 5–9 year olds (AAPC = 3.16%, 95%CI 1.03 to 5.34, *p* = 0.009) (Table 3).

## Discussion

This retrospective analysis using the ‘cascade of care’ analysis approach has revealed substantial room for improvement in the delivery of ear health services for Aboriginal children and young people living in remote NT communities. It identified poor performance in both ear health surveillance and management of OM, in the context of a persisting high prevalence of OM from the first few months of life. Surveillance data show that 70–80% of children aged under five years received at least one ear examination per six months, which means up to 30% did not received an ear examination and were at risk that OM would not be detected. Of those who were examined, around 40% had OM and of these an average of fewer than one in four were treated or followed-up in accordance with the local guidelines. Under-servicing leads to serious, long-term consequences for many children due to the conductive hearing loss caused by persisting OM or progression to tympanic membrane perforation and chronic suppurative OM [31], which increase the risk of hearing-related developmental delay and poorer educational and social outcomes [11–13].

Our findings have important implications for both control of OM and the provision of ear health services in remote NT communities. The limited surveillance and poor compliance with OM guidelines for Aboriginal and Torres Strait Islander children call for better policy and practice in both areas. More efforts are required to enhance the awareness of clinicians about the high risk of OM in Aboriginal children, that AOM rarely presents as sudden onset or pain, and does not resolve without intervention, emphasising the need for regular ear examinations, early detection, and appropriate treatment [2, 19, 32, 33]. This can be done through making it part of the service guideline to offer ear examination each time children visit remote clinics, in addition to an active age-appropriate surveillance program. Evidence to support recommendations on the timing of ear checks has very recently been published as a national consensus statement [34]. These should replace current consensus recommendations that vary from three-monthly from four months or age [35], to at-risk children three- to six-monthly [36], at immunisation visits [37], to scheduling six-monthly ear assessments for those not seen or who have missed a follow-up assessment [19]. The national consensus statement recommends for children not already being managed for ear and hearing problems that checks occur at least six-monthly, commencing at six months until four years of age, then at five years. Checks should be more frequent in high risk settings for children under two years, when acceptable to families, or in response to parent/carer concerns. In order for these measures to be effective, regular and more effective staff training to improve competence in performing ear and hearing examinations may also be required, especially given the persisting health service workforce shortage and high turnover rates in remote NT clinics [38]. Another example to support the need for better staff training is the Healthy Under 5 Kids (HU5K) program, a comprehensive well child health program implemented by NT Department of Health in remote communities. A total of ten clinic visits for child health assessment are scheduled under this program to occur at key ages of a child’s life, including five for the first year, two for the second year and one per year between ages two and four years. A recent evaluation report showed that between 2011 and 2016, ear examination (otoscopy) was performed in 33% of scheduled visits, no hearing screens were done, and tympanometry was rarely used (< 5%) while a diagnosis was made in 10% [39]. Currently, the Hearing for Learning Initiative is conducting a stepped-wedge cluster randomised controlled trial in remote NT communities to address the health service workforce shortfall. The study intervention involves training and employment for locally based, Aboriginal ear health facilitators to improve sustainability of culturally appropriate ear and hearing surveillance, and to facilitate delivery of evidence-based clinical care [21]. This research will also help understand the facilitators and barriers to accessing both primary and specialist ear and hearing health services.

Our study also found significant decreasing trends in OM prevalence in all age groups and a nearly 40% overall decrease during the study period. However, it is not known if there was a similar trend and an overall decrease in those who did not receive ear examinations, especially in the 5–9 and 10–16 years age groups in which high proportions of children did not receive an ear examination (on average, 40% and 60% respectively). It is also not known if the decrease was caused because, among those who had received ear examinations, there were increasing proportions of children without OM, replacing children with OM. This is a possible cause that cannot be ruled out, considering that 3 in 4 children who were diagnosed with OM were not treated or followed up in accordance with the local guidelines. Similar uncertainty also applies to the magnitude of the calculated OM prevalence per se. The average prevalence of OM was 53.1% in under 2-year-old children, which is far lower than previously reported high prevalence of 90% in a survey of 651 children (mean age 17.8 months) in 26 Top End communities conducted in 2010 to 2013 [2]. This PCIS analysis also found that about 12% had bilateral OME, which is also much lower than the 46% reported by the community survey. Several factors might have contributed to the differences, however, the major contributor is likely to be that the ear examinations recorded in the PCIS were performed by primary care clinicians whereas those from the community surveys were by specially trained research nurses using more advanced equipment. For example, in one recent survey [2], assessments were made using a tympanometer (Grason Stadler GSI 38), a LumiView (Welch Allyn) with Siegel’s speculum for pneumatic otoscopy, and a video-otoscope (Welch Allyn macroview or MedRx video-otoscopes). Such equipment is very rarely available in remote NT clinics where simple otoscopes are most commonly used and tympanometry or skills in pneumatic otoscopy are also rare. Guidelines recommend pneumatic otoscopy or otoscopy and tympanometry for ear assessments in this population due to the difficulty in differentiating no OM from OME. Further research is needed to ascertain if the observed prevalence and its decreasing trends reflect the actual epidemiology of OM in the whole population.

To the best of our knowledge, our study is the first in Australia to adopt the ‘cascade of care’ analysis approach in assessing the performance of ear health services in a setting with high OM prevalence and limited resources (as in remote NT Aboriginal communities). As demonstrated in this study, the ‘cascade of care’ approach is useful in quantitatively identifying specific ‘hot-spots’ requiring improvement in the whole spectrum of ear health service delivery. Additionally, we believe there is potential to use the statistics thus produced to formulate key performance indicators for ongoing assessment and monitoring of the quality of ear health services delivered at remote clinics. The other strength of this study is the use of unit record data linked with unique client identification numbers and the coverage of the data across different areas of the NT. The coverage means the representativeness of our data for the population was good. However,

One of the limitations of our study is that almost half of the remote Aboriginal communities in the NT are serviced by Aboriginal Medical Services and therefore not represented in our data. Aboriginal people have been reported to move between communities and access different remote clinics. This means some of those represented in the PCIS data used in this study might have valid ear health data recorded elsewhere, leading to missing data. The extent to which client mobility affects the cascade analysis is not known.

There are also other limitations. We had applied strict criteria in our search for services for ear examination and OM treatment and follow-up, and this might have led to certain degrees of under-counting. Anecdotal accounts have indicated that some clinicians (e.g. those not well versed in entering data in PCIS) tended to enter clinical information in fields other than the designated data fields for ear examination and medication (such as the free-text fields for progress notes). Further, as this study was conducted as a baseline study for the Hearing for Learning Initiative, it used the key primary and secondary indicators of the Initiative as the outcome measures and limited the analysis to ten 6-month periods over the study period. This approach could lose some valid outcomes that stretched across two periods. The Hearing for Learning Initiative investigators have a plan to conduct a separate study to analyse that same data but without the time period limits. The results may complement the findings of the current study.

## Conclusions

Our study found poor compliance in all steps of the cascade of care for ear health in remote Aboriginal communities in the NT. It also found decreasing trends in OM prevalence across all age groups, although the OM prevalence remained extremely high. To effectively reduce the prevalence of OM and its complications, it is crucial to improve ear health surveillance systems, diagnostic skills and confidence, and guideline compliance in OM management. Further research is required to understand the facilitators and barriers to Aboriginal children accessing and/or receiving ear health services for ear health surveillance and OM management at their local remote clinics. This study has demonstrated the utility of the cascade of care analysis in the evaluation of ear health services in remote settings.

## Data Availability

Menzies is the trial data custodian, managed by the Senior Data Manager. The HfLI Data Manager and Analyst, Program Manager and trial investigators had access to the data used in this study. The point of contact is the corresponding author of this manuscript, Jiunn-Yih Su. Funding partners did not have access to data.
